# Reversible endoscopic gastroduodenal bypass for the treatment of persistent duodenal leaks after failed surgical repair: a pilot feasibility study

**DOI:** 10.1055/a-2544-8507

**Published:** 2025-05-14

**Authors:** Kambiz Kadkhodayan, Saurabh Chandan, Artur Viana, Maham Hayat, Natalie Cosgrove, Mustafa A Arain, Deepanshu Jain, Abdullah Abassi, Sagar Pathak, Dennis Yang, Muhammad Khalid Hasan, Armando Rosales, Jay Redan, Shayan Irani

**Affiliations:** 1 440172 Center for Interventional Endoscopy, AdventHealth Orlando, Orlando, United States; 2 Gastroenterology, New York University, New York, United States; 3 Center for Interventional Endoscopy, Florida Hospital Orlando, Orlando, United States; 4 Gastroenterology and Hepatology, Virginia Mason Medical Center, Seattle, United States

## Abstract

**Background:**

Post-surgical leaks following surgical repair of acute duodenal perforations carry high mortality. Reversible endoscopic gastroduodenal bypass (REGB) is a novel procedure that helps divert the acid-rich gastric stream away from the affected duodenum to promote tissue healing at the ulcer site.

**Methods:**

REGB is a single-session, two-step procedure involving the creation of an endoscopic ultrasound-guided gastrojejunostomy using a lumen-apposing metal stent, followed by endosuturing and closure of the pylorus to achieve complete duodenal bypass. The outcomes of REGB and its reversal were prospectively evaluated in six patients with persistent post-surgical duodenal leaks.

**Results:**

REGB was technically successful in all six patients (100%) with no procedure-related adverse events. All patients resumed oral intake within 3 days, experienced significant reductions in surgical drain output, and were discharged. One patient with metastatic breast cancer did not undergo REGB reversal. Among five patients who underwent REGB reversal after a mean of 52.6 days, technical success was achieved in all (100%), with complete healing of duodenal ulcers, absence of leaks on fluoroscopy, and resumption of a solid food diet.

**Conclusion:**

REGB is a technically feasible, reversible, and minimally invasive alternative for managing post-surgical duodenal leaks. Further studies are needed to validate its safety and efficacy.

## Introduction

Duodenal perforation is a common surgical emergency with a mortality rate ranging from 4% to 30% in Western countries 1 . Common etiologies include peptic ulcer disease, abdominal trauma, complications from abdominal surgery or gastrointestinal procedures such as endoscopic retrograde cholangiopancreatography and endoscopic ultrasound (EUS) 2 3 . While primary surgical closure with an omental patch is the preferred treatment for simple duodenal perforations, data regarding the optimal management for large duodenal perforations remain limited. Reported strategies include duodenojejunostomy, pedicled grafts, pyloric exclusion with gastrojejunostomy and drain placement, gastric body partition, and in extreme cases, pancreaticoduodenectomy. Post-surgical complications such as perforation, bleeding, and postoperative duodenal leaks, in particular, are associated with significant mortality and often necessitate reoperation 4 5 . Challenges associated with a repeat operation in such patients include adhesions between abdominal viscera and the undersurface of the anterior abdominal wall, a condition widely known as “frozen abdomen,” poor tissue quality, and medical deconditioning especially in patients with multiple comorbidities and poor nutritional status. Given the high morbidity associated with surgical re-exploration, clinicians are increasingly considering endoscopic therapies as a viable alternative.

Duodenal diversion via gastrojejunostomy was originally conceived in the early 1900s. A simple, yet elegant technique of surgically excluding the duodenum was first reported by Vaughan et al. in 1977 6 . The operation consisted of primary repair of the duodenal defect, followed by closure of the pylorus via a gastrotomy that served as a site for gastrojejunal anastomosis. Pyloric exclusion offers a valuable approach in the management of duodenal perforations. While studies have reported mixed outcomes, with some reporting no mortality benefit compared with primary repair and others indicating increased complications or prolonged hospital stays, pyloric exclusion remains a viable approach to organ preservation. In cases where pyloric exclusion is not performed as part of the primary operation and a leak develops, surgical options for repair become limited, often necessitating more extensive and non-organ-sparing interventions 7 8 .

One of the advantages of enteric stream diversion following surgical duodenal leak repair, is that it may help facilitate mucosal healing by acting as a “protective barrier,” preventing the ulcer bed from exposure to gastric acid. This is particularly important in patients who are refractory to acid suppressive medical therapy such as proton pump inhibitors (PPIs). Such patients may not experience a true reduction in gastric pH following standard PPI therapy. In these patients, diversion of the acid-rich enteric stream into the jejunum may result in a higher pH in the duodenum, reduction of duodenal inflammation, promotion of granulation tissue formation, and ultimately restoration of the duodenal mucosal integrity 9 10 . Furthermore, the absence of mechanical pressure at the ulcer site caused by the ingested solid food that patients with stable fistulas are generally started on, may also promote healing.

The endoscopic equivalent of surgical pyloric exclusion, by means of the reversible endoscopic gastroduodenal bypass (REGB) procedure, is a single-session procedure involving the application of an EUS-guided gastrojejunostomy (EUS-GJ), resulting in partial diversion of the gastric stream toward the proximal jejunum, followed by endoscopic closure of the pylorus with the overstitch device, resulting in complete diversion of the antegrade gastric stream away from the duodenum 11 . This novel endoscopic approach offers a technically feasible and less morbid alternative to open surgical reintervention in patients with postoperative duodenal leaks. We describe our initial experience with six patients who successfully underwent REGB at our center.

## Methods

### Design

The study was conducted at a large tertiary referral center. Data regarding patient demographics, procedural details, and follow-up were prospectively collected.

### Patient characteristics

Six consecutive patients (four males, two females), aged 41–84 years, with acute perforated duodenal peptic ulcers were included. All patients experienced a persistent leak following primary surgical repair with an omental patch and high-dose PPI therapy. All patients underwent the REGB procedure at a median interval of 12.3 days (mean 14.5 days) following primary surgical repair.

### Procedure description

#### Procedure 1: REGB ( Fig. 1 )

**Fig. 1:**
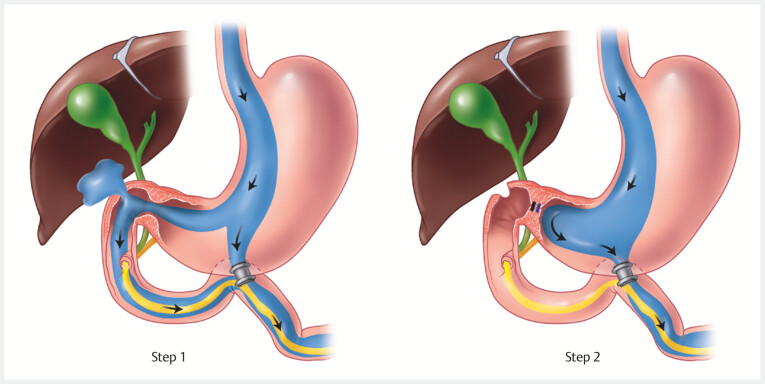
Illustration of both steps of the reversible endoscopic gastroduodenal bypass procedure. Step 1 – endoscopic ultrasound-guided gastrojejunostomy. Step 2 – endoscopic closure of the pylorus. Source: Elena S. Kakoshina.

**Fig. 2:**
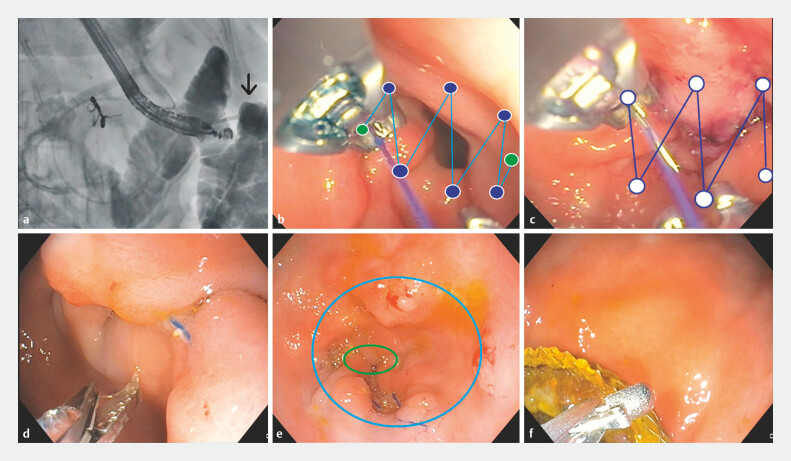
The two steps of the reversible endoscopic gastroduodenal bypass procedure. **a** Fluoroscopy image of procedure 1 (reversible endoscopic gastroduodenal bypass [REGB]) step 1: placement of lumen-apposing metal stent (LAMS). The image shows the LAMS being placed through the distal gastric body into a loop of proximal jejunum (black arrow). **b** Endoscopy image of procedure 1 (REGB) step 2: the first layer of transmural sutures is applied at the pylorus. The suture starts and ends at the antrum without directly engaging the pyloric ring (green dots). The remaining suture bites engage the pyloric ring (blue dots). **c** Endoscopy image of procedure 1 (REGB) step 2: the second layer of transmural sutures is applied 2–3 cm above the first layer. **d** Endoscopy image of procedure 2 (REGB reversal) step 1: cutting of the pyloric sutures using endoscopic scissors. **e** Endoscopy image after cutting the first layer of sutures (blue ring), exposing the second layer of sutures (green ring), which are subsequently cut using endoscopic scissors. **f** Endoscopy image showing the removal of the LAMS using rat tooth forceps with gentle backward traction.

**Step 1** Using a curvilinear echoendoscope, a suitable loop of proximal jejunum was identified. An EUS-GJ was then performed using the wireless EUS-guided gastroenterostomy simplified technique and a 20 × 10 mm lumen-apposing metal stent (LAMS) ( Fig. 2 **a** ) 11 .

**Fig. 3:**
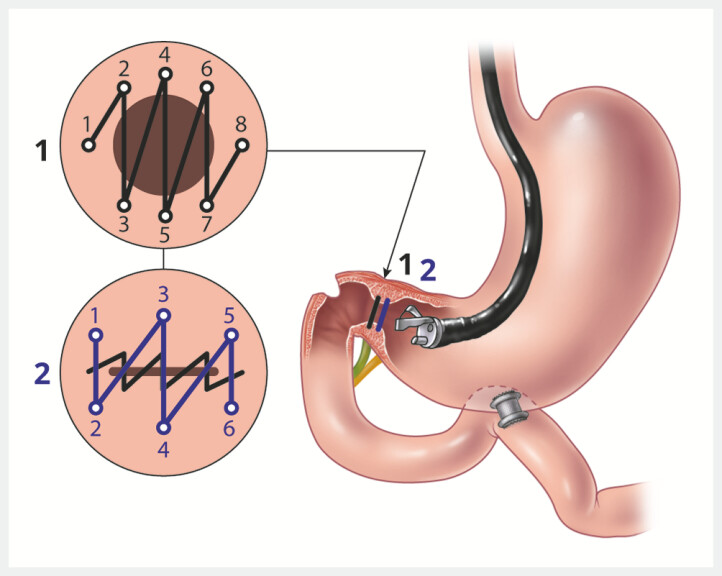
Illustration of the suturing technique used for closure of the pylorus. Source: Elena S. Kakoshina.

**Step 2** With the help of an endoscopic suturing device (Apollo OverStitch; Boston Scientific, Marlborough, Massachusetts, USA), transmural sutures were applied to the pyloric ring in a continuous fashion using a 2.0 polypropylene suture ( Fig. 2 **b** ). This was followed by the application of a second layer of transmural sutures at the antrum 2–3 cm above the first layer ( Fig. 2 **c** , Fig. 3 ).

**Fig. 4:**
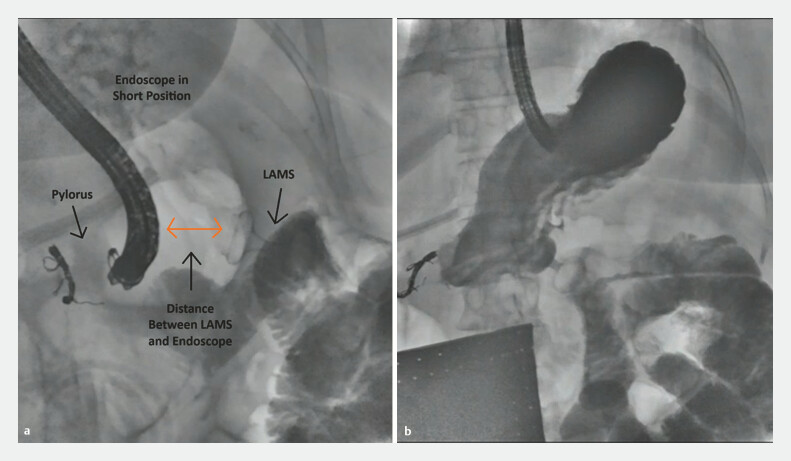
Fluoroscopy images. **a** The gastroscope is kept in a “short” position. The shaft of the scope is located away from the lumen-apposing metal stent (LAMS). **b** Complete bypass of the duodenum is confirmed by contrast injection. The injected water-soluble contrast flows through the LAMS into the proximal small bowel, with the patient in a reverse Trendelenburg position. No contrast passes through the pyloric sutures into the duodenum.

It is important to note that the pylorus was not de-epithelialized prior to suturing. This allows the sutures to be comfortably removed during the subsequent reversal procedure. Additionally, we were cautious not to apply excessive pressure on the newly placed LAMS while suturing the pylorus. This was accomplished by keeping the patient in a supine or partial left-lateral position, keeping the endoscope in the “short” position, and when needed, tilting the fluoroscopy bed in a direction whereby gravity helped to keep the endoscope away from the LAMS ( Fig. 4 **a** ). At the end of procedure 1, complete bypass of the duodenum was confirmed in all patients via contrast injection and fluoroscopy ( Fig. 4 **b** ).

Following the REGB procedure, patients were started on a diet and the surgical drain output was clinically monitored. Patients were scheduled for a reversal procedure after the surgical drain output had consistently decreased to below 30 mL in 24 hours.

#### Procedure 2: Reversal of the REGB procedure ( Fig. 5 )

The reversible endoscopic gastroduodenal bypass procedure and subsequent reversal procedure. Source for graphical illustrations: Elena S. Kakoshina.Video 1

**Fig. 5:**
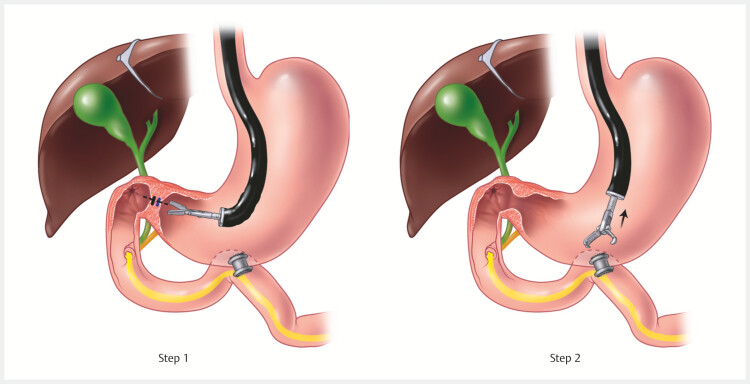
The reversal procedure. Source: Elena S. Kakoshina.

**Step 1** The polypropylene sutures were cut using standard endoscopic scissors ( Fig. 2 **d,e** ). This resulted in re-opening of the pylorus and restoration of the transpyloric stream. The duodenal peroration site was visually examined to ensure complete healing, and a persistent duodenal leak was excluded on fluoroscopy via intraluminal contrast injection, before proceeding to step 2.

**Step 2** Using rat tooth forceps and gentle backward traction, the LAMS was removed ( Fig. 2 **f** ). Following this, surgical drains were removed under fluoroscopy.

**Fig. 6:**
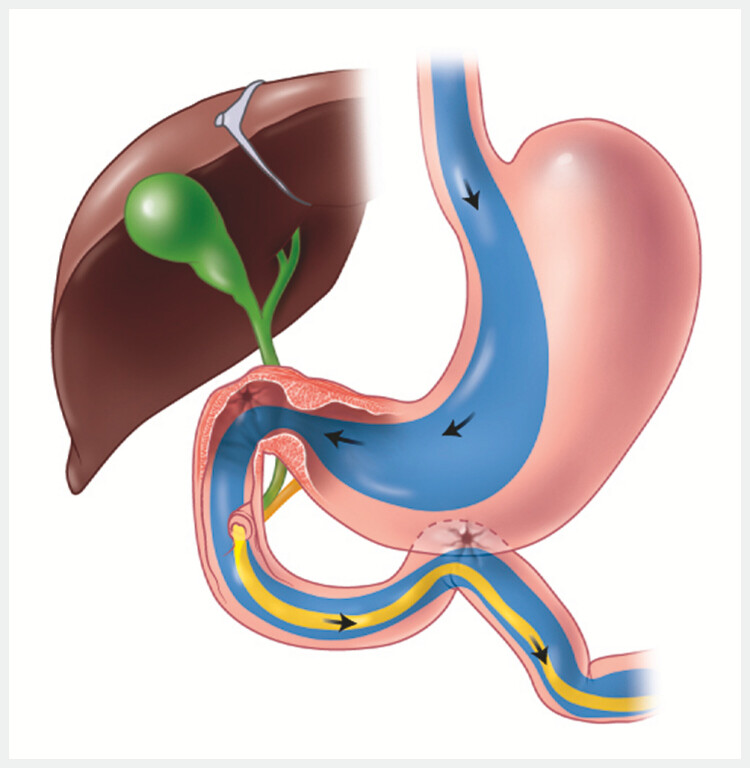
Complete reversal of the reversible endoscopic gastroduodenal bypass procedure and restoration of transpyloric flow. Source: Elena S. Kakoshina.

Completion of step 2, and spontaneous closure of the gastrojejunal fistula, resulted in complete restoration of the transpyloric stream, and reversal of the duodenal bypass ( Fig. 6 , Video 1 ).

### Outcomes assessed

Technical success was defined as completion of all planned steps of procedure 1 (REGB) and procedure 2 (reversal of REGB). Completion of duodenal bypass after procedure 1, and reversal of duodenal bypass after procedure 2 were confirmed via intraprocedural injection of water-soluble contrast and fluoroscopy. Clinical success for procedure 1 was defined as the ability to start an oral diet, significant decrease in the surgical drain output (to less than 50% of the pre-procedure output), and ability to discharge the patient from the hospital. Clinical success for procedure 2 was defined as complete healing of the duodenal ulcer, absence of a duodenal leak on fluoroscopy, and the ability to restart a solid food diet. Additional parameters such as procedure-related adverse events and body weight were also recorded.

## Results

REGB (procedure 1) was technically successful in all six patients (100%). There were no procedure-related adverse events. All six patients (100%) were started on an oral diet within 3 days of the procedure, experienced significant decreases in their surgical drain output, and were discharged from the hospital. After the first procedure, one patient with metastatic breast cancer who elected for hospice care chose not to undergo procedure 2, and died of unrelated causes 5 months later.

REGB reversal (procedure 2) outcomes were assessed in the remaining five patients. The mean time to bypass reversal was 52.6 days (median 50 days, range 42–72 days). Technical success was achieved in all five patients (100%), with complete healing of the duodenal ulcers on endoscopy, absence of a duodenal leak on fluoroscopy, and the restarting of a solid food diet (see **Table 1s** in the online-only Supplementary material). The average change in body weight between procedure 1 (REGB) and procedure 2 (REGB reversal) was –10.7 kg (range +2 kg to –14 kg). Three patients had a repeat endoscopy after 3–6 months, which revealed spontaneous closure of the gastroenteric fistula.

## Discussion

Our initial single-center experience of six patients with acute duodenal perforations who developed post-surgical leaks highlights the potential of REGB, an endoscopic therapeutic alternative to surgical reintervention. The procedure can be performed in a single session with technical ease and is associated with high clinical success. Key advantages of this novel approach are that it is minimally invasive, avoids the need for a repeat surgery that can be highly morbid and technically challenging, is organ sparing, and perhaps most importantly, it is reversible and restores normal anatomic and physiologic function after resolution of the duodenal leak.

Omental patch repair (omentopexy) via laparotomy remains a standard intervention for perforated duodenal ulcers, but the incidence of postoperative leaks remains high. Studies on patients with perforated peptic ulcers treated by omental patch repair have estimated leak rates of 4%–7.6%, with mortality ranging between 29.4% and 55.6% in patients who experience a leak 12 13 . Several factors have been attributed to the incidence of leaks after surgery for duodenal ulcer perforations, such as patient factors (age, shock on presentation, malnutrition, pre-existing comorbidities, low hemoglobin, and low serum albumin), poor surgical technique, large perforation size, delay in seeking medical attention, and delay in diagnosis and surgery 14 . Given the limitations and high morbidity associated with surgical re-exploration, especially among high-risk individuals, REGB may be a suitable therapeutic modality in such patients.

A large study of over 700 patients with a perforated peptic ulcer who underwent surgery with either a laparoscopic or open laparotomy approach reported that 17.1% patients had reoperative surgery, the most common reason for which was reperforation (5.9%), followed by wound dehiscence (4.7%) 15 . This is of particular importance as surgical reintervention in patients with duodenal leaks following surgical repair may be particularly challenging given the possibility of a “frozen abdomen,” characterized by the presence of dense adhesions among abdominal viscera and poor nutritional status 16 . In this regard, an endoscopic approach utilizing REGB completely circumvents the operative field and may be a potentially safer option.

It is important to note that patients in our series lost a significant amount of weight between the first and second procedure. Potential reasons for this weight loss could be metabolic stress, catabolic state related to starvation, calorie restriction, and exclusion of absorptive mucosa in the duodenum. Of note, the patients regained almost all the lost weight at 3 months’ follow-up after the bypass was reversed.

Our study has several limitations that warrant consideration. First, our report is a pilot feasibility study with a small sample size that limits generalizability of the findings. Second, all REGB procedures were performed at a tertiary referral center by an experienced advanced endoscopist (K.K.) and may not reflect outcomes that are achievable in community or less-specialized settings. Third, we were not able to systematically assess protein malnutrition or other nutritional deficiencies that may result from duodenal exclusion, which could impact patient recovery. Fourth, the follow-up period in this study was short, preventing assessment of long-term outcomes such as stricture formation and quality of life scores. Fifth, the majority of patients described here had perforations and leaks located in the duodenal bulb and it is unclear whether the duodenal bypass will be helpful in perforations of the distal duodenum that receives a continuous flow of bile and pancreatic juices. Sixth, the procedure requires additional training in therapeutic endoscopy, interventional EUS, and endoscopic suturing. Technical feasibility and outcomes of REGB may vary significantly with operator experience, and the steep learning curve associated with this procedure may hinder widespread adoption. Finally, the study did not evaluate the cost-effectiveness of REGB compared with surgical reintervention. These limitations highlight the need for larger, multicenter studies with longer follow-up to validate the findings and assess the broader applicability of the procedure.

### Conclusion

REGB is a technically feasible and clinically successful therapeutic intervention for patients with postoperative duodenal bulb leaks. Further larger studies are needed to evaluate the safety, efficacy, and long-term outcomes of this procedure.

## Supplementary Material

Supplementary Material Zusatzmaterial
