# Supplementary material for: Reversible endoscopic gastroduodenal bypass for the treatment of persistent duodenal leaks after failed surgical repair: a pilot feasibility study
Source: Endoscopy. 2025 May 14;57(8):892–8. doi: 10.1055/a-2544-8507 (PMC12307041; doi:10.1055/a-2544-8507)
Supplement: Supplementary file 2 — Supplementary Material [file 10-1055-a-2544-8507_25802419.pdf]

Supplementary material

Reversible endoscopic gastroduodenal bypass for the treatment of persistent duodenal leaks after failed surgical repair: a pilot feasibility study

Kambiz Kadkhodayan, Saurabh Chandan, Artur Viana, Maham Hayat, Natalie Cosgrove, Mustafa A. Arain, Deepanshu Jain, Abdullah Abassi, Sagar Pathak, Dennis Yang, Muhammad K. Hasan, Armando Rosales, Jay Redan, Shayan Irani

Thieme

Table 1s Patient outcomes after REGB and REGB-reversal.

	Patient 1	Patient 2	Patient 3	Patient 4	Patient 5	Patient 6
**Age**	68	82	72	76	84	41
**Sex**	M	F	M	M	F	M
**Indication**	Duodenal bulb ulcer perforation, post-op leak. Bile duct injury – required ERCP with stent	Duodenal bulb ulcer perforation, post-op leak	Duodenal (second part) ulcer perforation with abscess.	Duodenal bulb ulcer perforation, post-op leak.	Duodenal bulb ulcer perforation, post-op leak.	Duodenal bulb perforation with post-op leak. Concern for bile duct leak, required ERCP with stent
**Time from surgical repair to REGB procedure**	20 days	15 days	7 days	14 days	15 days	3 days
**Technical Success of REGB procedure**	Yes	Yes	Yes	Yes	Yes	Yes
**Adverse events related to REGB procedure**	No	No	No	No	No	No
**Clinical success of REGB procedure**	Yes	Yes	Yes	Yes	Yes	Yes
**Time from REGB to reversal procedure**	50 d	49 d	72 d	50 d	NA ++	42 d
**Technical success of reversal procedure**	Yes	Yes	Yes	Yes	NA ++	Yes
**Adverse events related to reversal procedure**	No	No	No	Yes +	NA ++	No
**Clinical success of reversal procedure**	Yes Resolution of ulcer and bile leak	Yes	Yes	Yes	NA ++	Yes Resolution of ulcer and bile leak
**Weight change between the REGB and reversal procedure (Kg)**	+2 kg	- 12.9 kg	- 14 kg	-9 kg	NA++	- 0.6 kg

+ Patient 4 developed a mucosal bridge at the pylorus. This was managed endoscopically with a scissor type electrosurgical knife. Of note, the mucosal bridges developed around a prior pyloric channel ulceration.

++ Patient 5 was discharged to hospice due to metastatic breast cancer. She opted not to have the reversal procedure and died of unrelated causes 5 months later.
